# Sex Hormone Regulation of Proteins Modulating Mitochondrial Metabolism, Dynamics and Inter-Organellar Cross Talk in Cardiovascular Disease

**DOI:** 10.3389/fcell.2020.610516

**Published:** 2021-02-11

**Authors:** Shannon Lynch, James E. Boyett, M. Ryan Smith, Samantha Giordano-Mooga

**Affiliations:** ^1^Biomedical Sciences Program, Graduate School, University of Alabama at Birmingham, Birmingham, AL, United States; ^2^Biomedical Sciences Program, Department of Clinical and Diagnostic Science, University of Alabama at Birmingham, Birmingham, AL, United States; ^3^Division of Pulmonary, Allergy and Critical Care Medicine, Emory University, Atlanta, GA, United States

**Keywords:** sexual dimorphism, cardiovascular disease, estrogen, testosterone, mitochondria

## Abstract

Cardiovascular disease (CVD) is the leading cause of death in the U.S. and worldwide. Sex-related disparities have been identified in the presentation and incidence rate of CVD. Mitochondrial dysfunction plays a role in both the etiology and pathology of CVD. Recent work has suggested that the sex hormones play a role in regulating mitochondrial dynamics, metabolism, and cross talk with other organelles. Specifically, the female sex hormone, estrogen, has both a direct and an indirect role in regulating mitochondrial biogenesis via PGC-1α, dynamics through Opa1, Mfn1, Mfn2, and Drp1, as well as metabolism and redox signaling through the antioxidant response element. Furthermore, data suggests that testosterone is cardioprotective in males and may regulate mitochondrial biogenesis through PGC-1α and dynamics via Mfn1 and Drp1. These cell-signaling hubs are essential in maintaining mitochondrial integrity and cell viability, ultimately impacting CVD survival. PGC-1α also plays a crucial role in inter-organellar cross talk between the mitochondria and other organelles such as the peroxisome. This inter-organellar signaling is an avenue for ameliorating rampant ROS produced by dysregulated mitochondria and for regulating intrinsic apoptosis by modulating intracellular Ca^2+^ levels through interactions with the endoplasmic reticulum. There is a need for future research on the regulatory role of the sex hormones, particularly testosterone, and their cardioprotective effects. This review hopes to highlight the regulatory role of sex hormones on mitochondrial signaling and their function in the underlying disparities between men and women in CVD.

## Cardiovascular Disease and Sex Steroid Signaling

Cardiovascular disease (CVD) is modulated by mitochondrial dysfunction, calcium handling, aging, etc., which are reviewed in detail in the corresponding reviews (Khoury et al., 1992; Sandstede et al., 2000; Berridge, 2003; Lou et al., 2012; Keller and Howlett, 2016; Ventura-Clapier et al., 2017; Virani et al., 2020). Sex disparities in the cardiovascular system including heart size, body size, adipose deposition, etc. have been linked to variations in CVD risk and rates. In this review, we will focus on sex hormone driven differences in CVD. In general, women express higher levels of estrogen and estrogen receptors (ERs) than men, while men express higher levels of testosterone and androgen receptors (ARs) than women; as both sexes age, there is a decrease in the predominant sex hormone. While the primary sex hormone decreases, there is a concurrent increase in estrogen in men and an increased ratio of testosterone to estrogen in women (Araujo and Wittert, 2011; Bowling et al., 2014; Zhao et al., 2018).

Currently, there are conflicting results on the role of testosterone in CVD, but it is documented that the decrease in testosterone in men with age and the higher ratio of testosterone to estrogen in post-menopausal women may be linked to increased CVD incidence (Freeman et al., 2010; Elagizi et al., 2018). Human studies have shown confounding results regarding the cardioprotective effects of menopausal hormone therapy to increase estrogen levels in women during the post-menopausal period (Stampfer et al., 1991; Rossouw et al., 2002; Hodis and Mack, 2014). The stark decrease in estrogen levels in the post-menopausal period have also been linked to obesity and metabolic syndrome incidence (Araujo and Wittert, 2011; Ziaei and Mohseni, 2013; Bowling et al., 2014; Moore et al., 2017; Zhao et al., 2018; Terrazas et al., 2019). The sex hormones also regulate differences in metabolism, specifically in regards to fat accumulation and body shape between men and women, which are known modulators of CVD risk (Bjorntorp, 1997; Fui et al., 2014; Mercado et al., 2015; Van Pelt et al., 2015; Leeners et al., 2017; Terrazas et al., 2019). The metabolic differences associated with changes in hormone status—which are influenced by a plethora of factors including sex chromosomes, gene expression and regulation, and epigenetics—are key to understanding CVD disparities between men and women (Ventura-Clapier et al., 2019). This review will focus on sex hormone signaling and its potential cardioprotective effects, discuss controversial findings regarding sex hormone signaling, and highlight the need for further research to create efficacious and sex-specific CVD treatments.

To elucidate the roles of estrogen and testosterone in CVD, it is essential to understand the roles of their associated receptors, including estrogen receptor alpha (ERα), estrogen receptor beta (ERβ), G protein coupled estrogen receptor (GPER/GCPR30), and ARs. The regulation of estrogen and androgen receptor expression is challenging to study, as they are sex-, age-, cell type-, and organelle-specific (Lizotte et al., 2009; Dart et al., 2013; Bowling et al., 2014; Hutson et al., 2019). At the cellular level, it has been shown that varying cell types express different levels of sex hormone receptors, highlighting that each organ system may have differential sexual dimorphic regulation (Erlandsson et al., 2001; Deroo and Korach, 2006; Levin, 2009; Dart et al., 2013; Mahmoodzadeh and Dworatzek, 2019; Ventura-Clapier et al., 2019). Within the cell, sex hormone receptors can be found in a variety of locations including the cell membrane, nucleus, mitochondria, and endoplasmic reticulum, although, again, these locations vary depending upon the cell type (Levin, 2009; Lizotte et al., 2009; Luo and Kim, 2016; Pedernera et al., 2017). In cardiomyocytes, the hormone receptors are expressed at different locations on various organelles; for example, both ERα and ERβ have been found to be localized to the mitochondria, while GPER has been localized to both the cell membrane and the endoplasmic reticulum (Lizotte et al., 2009; Luo and Kim, 2016; Zimmerman et al., 2016; Pedernera et al., 2017; Gourdy et al., 2018; Ventura-Clapier et al., 2019). While receptor expression within the cardiovascular system is known, further studies understanding how the ERs and ARs change based on age and hormone status is needed.

The mitochondrion plays an integral role in the production of the steroid hormones, as it is the site wherein the first step of sex hormone synthesis occurs (Miller, 2013). These same sex hormones are implicated in regulating mitochondrial dynamics and function. Cholesterol is the building block for the steroid hormones, specifically the C27-steroid cholesterol, which enters the mitochondria through the steroidogenic acute regulatory protein where the cytochrome P450 enzyme, CYP11A1, produces pregnenolone; pregnenolone can subsequently be transported back into the cytosol and converted, through a series of enzymatic steps, into either estrogen (estradiol) or testosterone (Hu et al., 2010; Miller and Bose, 2011; Samavat and Kurzer, 2015). The enzyme aromatase converts testosterone into estradiol, and recent studies have found elevated aromatase correlates with metabolic dysfunction in women (Araujo and Wittert, 2011; Iyengar et al., 2017). It has also been shown that adipose tissue is the primary producer of estrogen in post-menopausal women, adding to the complexity of estrogen signaling (Cleland et al., 1985; Chen and Madak-Erdogan, 2018). The differences in serum estrogen and testosterone levels can greatly influence cellular processes and adipose deposition, which can modulate CVD risk.

Testosterone and dihydrotestosterone activate the nuclear AR, which regulates transcription of genes located near androgen response elements or via a DNA binding independent pathway to activate ERK, Akt, and MAPK pathways (Benten et al., 1999b; Davey and Grossmann, 2016). Plasma membrane associated ARs play an important role in calcium (Ca^2+^) signaling, in addition to influencing endoplasmic reticulum signaling and apoptosis (Benten et al., 1999a,b; Segawa et al., 2002; Davey and Grossmann, 2016; Azhary et al., 2018). AR and androgen hormones are also essential for the development and normal physiology of the cardiovascular system (Ikeda et al., 2005). In comparison, the classical pathway of ERα activation involves its association with heat shock proteins within the cytosol; once estrogen binds ERα and/or ERβ, they can dimerize and translocate to the nucleus to activate transcription via the estrogen response element (Levin, 2009). ERα has also been shown to induce signaling cascades —including Akt, PKA and ERK1/2, and eNOS— through a membrane-initiated sequence whereby a post-translationally modified pool of ERα is localized near the plasma membrane due to an interaction with caveolin 1 (Levin, 2009; Yasar et al., 2017). After estrogen binds the receptor, it induces additional signaling pathways. ERα and ERβ are found on the plasma membrane, as both homo- and heterodimers, and expression is differential based on cell type, as previously mentioned (Li L. et al., 2003; Levin, 2009; Bowling et al., 2014). ERβ has been shown to localize to the mitochondria in cardiomyocytes of both humans and rodents, and has been proposed to play a role in mitochondrial integrity (Yang et al., 2004). GPER, a membrane-bound estrogen receptor, induces cAMP, IP3, Ca^2+^, and the MAPK/ERK pathways (Aronica et al., 1994; Improta-Brears et al., 1999; Filardo et al., 2000). Unlike ERα and ERβ expression, which are strictly regulated by estrogen levels and decrease in the post-menopausal period, GPER levels appear to be unaffected by circulating estrogen levels induced by menopause, but may fluctuate with estrous cycle (Cheng et al., 2014; Zimmerman et al., 2016). Data also suggests that GPER activation is protective after a vascular injury in ERα and ERβ KO mice, and can regulate mitochondrial function and biogenesis in ovariectomized mice (Bowling et al., 2014; Sbert-Roig et al., 2016; Mahmoodzadeh and Dworatzek, 2019). Therefore, more research is needed to determine the expression and role of GPER in preventing CVD injury.

## Cellular Metabolism

Mitochondria play a crucial role in many molecular pathways and cellular bioenergetics. Mitochondria comprise about 35% of the entire cell volume in cardiomyocytes, making their function even more crucial to proper cardiovascular function (Dedkova and Blatter, 2012; Consolini et al., 2017). The mitochondrion contains its own small genome, encoding 37 mitochondrial proteins which are translated in the mitochondria, while the remaining proteins and RNAs are encoded by the nuclear genome (Lee and Han, 2017). Since the majority of mitochondrial proteins arise from nuclear transcription, cross talk between the mitochondria and the nucleus is imperative for effective metabolism and function. Mitochondrial proteins and their precursors are transported from cytosolic ribosomes and the endoplasmic reticulum into the mitochondria and then integrated with mitochondrially-derived proteins via sorting, assembly, and importing machinery (Pfanner and Meijer, 1997; Ellenrieder et al., 2015, 2017; Doan et al., 2020). This, in conjunction with the formation of phospholipid precursors by the endoplasmic reticulum for the mitochondria, such as cardiolipin, shows the importance of cross communication for proper mitochondrial function, as well as modulation by hormones (Ellenrieder et al., 2017; Pozdniakova et al., 2018; Acaz-Fonseca et al., 2020).

Mitochondria utilize a variety of energy sources including glucose, fatty acids, and amino acids to produce reducing equivalents for mitochondrial respiration and ATP production, which is essential in cardiac tissue. Developing cardiomyocytes prefer glucose as their energy source, whereas adult cardiomyocytes prefer fatty acids; recent studies suggest that alteration of energy sources in cardiomyocyte metabolism can contribute to CVD progression (Piquereau et al., 2010, 2012; Krzywanski et al., 2011; Martin et al., 2014; Siasos et al., 2018). Mitochondrial metabolism produces reactive oxygen species (ROS), which are increased in mitochondrial dysfunction and prevalent in CVD (Wang and Zou, 2018). To reduce ROS, antioxidant proteins, including superoxide dismutase 1 (SOD1), SOD2, and glutathione peroxidase (GPx) can be upregulated (Table 1). In the heart, estrogen and testosterone have been shown to increase these antioxidant enzymes, and may act in a cardioprotective manner (Barp et al., 2002; Strehlow et al., 2003; Zhang et al., 2011; Lee et al., 2012; Liu H. et al., 2014; Liu Z. et al., 2014; Redmann et al., 2014; Pozdniakova et al., 2018; Mahmoodzadeh and Dworatzek, 2019; Ventura-Clapier et al., 2019; Asmis and Giordano-Mooga, 2020; Casin and Kohr, 2020). In the vasculature, GPER has been shown to modulate ROS through downregulation of oxidative stress proteins such as NADPH oxidase 4 (NOX4), Prostaglandin-endoperoxide synthase 2 (PTGS2), and GPx1 in addition to upregulating antioxidant proteins such as SIRT3 and GSTK1 (Wang H. et al., 2018; Ogola et al., 2019). Additionally, it has been shown that GPER downregulates an essential autophagy protein light chain 3—LC3I/LC3II—via upregulation of PTEN-induced putative kinase 1 (PINK1), and a concurrent decrease in mitochondrial parkin localization indicating a decrease in mitophagy (Feng et al., 2017). GPER activation, through mitochondrial and lysosomal cross talk mechanisms, is an important mitigator of ROS in CVD (Lee et al., 2012; Feng et al., 2017). These same antioxidant proteins have been associated with peroxisomal function and are key players in the peroxisome’s regulation of intracellular ROS levels (Schrader and Fahimi, 2006; Wang Q. et al., 2015). Because ROS regulation is critical in metabolic homeostasis and mitochondrial dynamics, perturbation of these processes often leads to increased risk of pathological outcomes, such as CVD (Krzywanski et al., 2011; Siasos et al., 2018). Hence, understanding how mitochondrial dynamics and cross talk are impacted by ROS and modulated by sex hormones is critical in elucidating the mechanisms underlying CVD.

**TABLE 1:** Impact of sex hormones on cardiac cell protein expression.

Protein/channel	Model	Testosterone (including DHT) regulation	Estrogen (including estradiol, estrone, and estrogen) regulation
ERα	(Lizotte et al., 2009) CD1 mice		Timing specific regulation
	(Park et al., 2017) Human skeletal muscle		Upregulated
	(Bowling et al., 2014) ERα KO and WT mice		Regulates expression
ERβ	(Lizotte et al., 2009) CD1 mice		No regulation
	(Park et al., 2017) Human skeletal muscle		Upregulated
	(Bowling et al., 2014) ERβ KO and WT mice		Regulates expression
GPER	(Wang H. et al., 2018) GPER-KO mice		
AR	(Lizotte et al., 2009; Pedernera et al., 2017) CD1 mice		
	(Kerkhofs et al., 2009; Huang et al., 2016) ARKO and ARKI mice		
	(Hanke et al., 2001) Rabbit aorta	Upregulation	
	(Dart et al., 2013) ARE-Luc knock-in mice	Upregulation	No regulation
	(Marsh James et al., 1998) Rat cardiomyocytes	Upregulation	No regulation
Unspecified SOD	(Zhang et al., 2011) Tfm mice	Upregulated	Upregulated
	(Barp et al., 2002) Wistar rats		Upregulated
	(Cruz-Topete et al., 2020) Mice cardiomyocytes	Upregulated	
SOD1	(Strehlow et al., 2003) Rat VSMCs		Upregulated
SOD2	(Strehlow et al., 2003) Rat VSMCs		Upregulated
	(Lone et al., 2017) MCF-7		Upregulated
	(Liu Z. et al., 2014) HAECs		Upregulated
	(Liu H. et al., 2014) Rat cardiomyocytes		Increased activity
GPx	(Zhang et al., 2011) Tfm mice	Upregulated	
	(Cruz-Topete et al., 2020) Mouse cardiomyocytes	Upregulated	
nNOS	(Casin and Kohr, 2020) Mouse cardiomyocytes		Upregulated
eNOS	(Casin and Kohr, 2020) Mouse cardiomyocytes		Upregulated
NOX4	(Wang H. et al., 2018) GPER-KO mice		Downregulated
	(Cruz-Topete et al., 2020) Human sex studies	Inconclusive	Inconclusive
	(Ogola et al., 2019) VSMCs GPER-KO mice		Downregulated
PTGS2	(Wang H. et al., 2018) GPER-KO mice		Downregulated
SIRT3	(Lone et al., 2017) MCF-7		Upregulated
	(Wang H. et al., 2018) GPER-KO mice		Upregulated
GSTK1	(Wang H. et al., 2018) GPER-KO mice		Upregulated
K^+^ATP Channel	(Sakamoto and Kurokawa, 2019) Rat cardiomyocytes	Opens channels during reperfusion, cardioprotective	Opens channels during reperfusion, cardioprotective
	(Gao et al., 2014) SUR_2_KO mice		Regulated, cardioprotective
	(Er et al., 2004) Sprague-Dawley rats cardiomyocytes	Activated, cardioprotective	
	(Ballantyne et al., 2013) H9c2 cells	Upregulates expression	Upregulates expression
SERCA	(Witayavanitkul et al., 2013) ORX mice cardiomyocytes	Increases activation	
			(Hill and Muldrew, 2014) Upregulates expression
PGC-1α	(Witt et al., 2008) AC16 cell line		Upregulation
	(Klinge, 2008) MCF-7 and H1793 cell lines		Upregulation via NRF-1
	(Klinge, 2008) Mouse cardiac tissue		Upregulation
	(Park et al., 2017) Human skeletal muscle		Upregulation
	(Wang F. et al., 2015) Wistar rat cardiomyocytes	Upregulates via AMPK	
Drp1	(Martin et al., 2014) PGC-1α and PGC-1β DKO mice		Upregulated*
	(Sastre-Serra et al., 2013) MDA-MB-231 cells		Upregulated
	(Sastre-Serra et al., 2013) T47D cells		Upregulated
	(Sastre-Serra et al., 2013) MCF-7 cells		No Effect
	(Capllonch-Amer et al., 2014) 3T3-L1 adipocytes	Downregulated	Upregulated
	(Lee et al., 2020) jLNCaP Cells	Upregulated	
Mfn1	(Martin et al., 2014) PGC-1α and PGC-1β DKO mice		Upregulated*
	(Papanicolaou et al., 2012) Mouse cardiomyocytes		Upregulated*
	(Sastre-Serra et al., 2013) MDA-MB-231 cells		Upregulated
	(Sastre-Serra et al., 2013) T47D cells		Upregulated
	(Sastre-Serra et al., 2013) MCF-7 cells		Upregulated
	(Capllonch-Amer et al., 2014)3T3-L1 adipocytes	Upregulated	No Effect
	(Lee et al., 2020) LNCaP cells	No Effect	
Mfn2	(Martin et al., 2014)PGC-1α and PGC-1β DKO mice		Upregulated*
	(Papanicolaou et al., 2012) Mouse cardiomyocytes		Upregulated*
	(Sastre-Serra et al., 2013) MDA-MB-231 cells		Upregulated
	(Sastre-Serra et al., 2013) T47D cells		Upregulated
	(Sastre-Serra et al., 2013) MCF-7 cells		Upregulated
	(Lee et al., 2020) LNCaP cells	No Effect	
Opa1	(Martin et al., 2014) PGC-1α and PGC-1β DKO mice		Upregulated*
	(Sastre-Serra et al., 2013) MDA-MB-231 cells		Upregulated
	(Sastre-Serra et al., 2013) T47D cells		Upregulated
	(Sastre-Serra et al., 2013) MCF-7 cells		Upregulated
	(Capllonch-Amer et al., 2014) 3T3-L1 adipocytes	No Effect	Upregulated
	(Lee et al., 2020) LNCaP cells	No Effect	
Fis1	(Martin et al., 2014) PGC-1α and PGC-1β DKO mice		Upregulated*
	(Sastre-Serra et al., 2013) MDA-MB-231 cells		No effect
	(Sastre-Serra et al., 2013) T47D cells		No effect
	(Sastre-Serra et al., 2013)MCF-7 cells		Downregulated
	(Capllonch-Amer et al., 2014) 3T3-L1 adipocytes	Downregulated	No effect
	(Lee et al., 2020) LNCaP cells	No effect	
PINK1	(Feng et al., 2017) Sprague–Dawley rats		Upregulated
LC3I/LC3II	(Feng et al., 2017) Sprague–Dawley rats		Downregulated
	(Papanicolaou et al., 2012) Mouse cardiomyocytes		Downregulated*

Summary of estrogen and testosterone regulation of antioxidant proteins, mitochondrial dynamics, intracellular Ca^2+^, and inter-organellar cross talk, both directly and indirectly.*Proteins indirectly regulated by the sex hormones via PGC-1α.

## Mitochondrial Biogenesis and Dynamics

Under normal conditions, mitochondrial turnover in the adult heart occurs every 2 weeks (Dorn et al., 2015). Mitochondrial dynamics is the process of mitochondrial growth and division designed to maintain homeostasis by utilizing the fission, fusion, mitophagy, and recycling processes during growth and development as well as under environmental stressors such as ischemia, hypoxia, or oxidative stress (Dorn et al., 2015; Dorn, 2016). Recent studies have shown that regulating mitochondrial homeostasis is crucial in mitigating CVD disruption of these processes which has been previously to worse pathological outcomes or increased death (Jornayvaz and Shulman, 2010; Sun et al., 2013; Redmann et al., 2014). Peroxisome proliferator-activated receptor gamma coactivator 1-alpha (PGC-1α) is a transcriptional coactivator protein crucial for maintaining homeostasis of this organelle by targeting genes involved in electron transport chain (ETC) and apoptotic signaling (Liang and Ward, 2006; Lai et al., 2008; Wang F. et al., 2015). AMP-activated protein kinase (AMPK) is an upstream regulator of PGC-1α involved in energy homeostasis and mitochondrial biogenesis and has been shown to be regulated through the sex hormones (Jornayvaz and Shulman, 2010; Varanita et al., 2015; Wang F. et al., 2015; Park et al., 2017; Hevener et al., 2020). In cardiomyocytes, activated estrogen receptors found on the cell membrane can upregulate PGC-1α activity, thereby regulating ATP synthesis, substrate oxidation, and phosphate transfer (Klinge, 2008; Witt et al., 2008). PGC-1α can also target genes that regulate metabolism in the heart, such as estrogen-related receptor alpha (ERRα), which protects against stressors of CVD (Huss et al., 2004; Martin et al., 2014; Li H. et al., 2015). While estrogen does not directly bind to ERRα, it activates genes regulated to mitochondrial biogenesis, and further studies are needed to understand the sexually dimorphic regulation (Horard and Vanacker, 2003). These data indicate a crucial signaling role for estrogen in the maintenance of mitochondria.

Estrogen has also been shown to activate peroxisome proliferator-activated receptor α (PPARα), a partner of PGC-1α, which functions to transcriptionally regulate fatty acid metabolism in the heart. Activation of PPARα induces the expression of Pex genes leading to peroxisomal biogenesis, while simultaneously inducing the expression of mitochondrial fusion and fission proteins mitofusin 1 (Mfn1), mitofusin 2 (Mfn2), dynamin-related protein-1 (Drp1), and fission protein 1 (Fis1) (Table 1; Bagattin et al., 2010; Papanicolaou et al., 2012; Schrader et al., 2012; Martin et al., 2014; Varanita et al., 2015). Increased number of peroxisomes in conjunction with sustained mitochondrial integrity increases β-oxidation of long chain fatty acids and fatty acid-induced cellular respiration. It has been further suggested that these tissues upregulate PGC-1α in response to increased lipid intake, acting as a compensatory mechanism for high fat diets and metabolic dysregulation. This co-regulation of peroxisomal and mitochondrial biogenesis has been established in brown adipose tissue, liver and skeletal muscle (Huang et al., 2017, 2019; Hevener et al., 2020). Work has yet to be done showing the proliferation of peroxisomes in cardiac tissue, but findings in other tissues is highly suggestive of the need for future research in this area.

PPARα KO mice have severely impaired cardiac function due to lipid accumulation and hypoglycemia which causes death in all males but only 25% of females; however, pretreatment of β-estradiol in males with ablated PPARα survived, implicating estrogen signaling as a crucial mechanism for cardiac metabolism (Nöhammer et al., 2003). Estrogen plays a crucial role in cardiac lipid metabolism for both males and females *in vivo* (Djouadi et al., 1998). This data, again, highlights the importance of estrogen signaling in cardiac metabolism.

Many cardiovascular pathologies have notable alterations in mitochondrial network morphology. Mitochondrial fission is a process by which mitochondria alter their physical structure; symmetrical division for replication or asymmetrical division to remove damaged organelle components (Shirihai et al., 2015). Asymmetrical fission acts as quality control for damaged mitochondria resulting in fragmentation, which can be utilized for selective mitophagy (Ong et al., 2010; Shirihai et al., 2015). While both processes serve as protective mechanisms for cellular damage and apoptosis through the mitochondria, the mechanisms of activation via other cellular organelles are different. In mitochondrial fusion, the mitochondria fuse with other organelles to repair and regenerate, as opposed to mitochondrial fission, where DNA replication is upregulated in response to mitochondrial damage, inhibiting cytochrome c release and corresponding apoptosis (Chen et al., 2012; Varanita et al., 2015; Dorn, 2016; Hevener et al., 2020). Major proteins associated with fission and fusion include Drp1, Fis1, Mfn1 and Mfn2, and the optic atrophy-1 protein (Opa1). Drp1 is recruited to the outer mitochondrial matrix (OMM) and has been shown to interact with the endoplasmic reticulum, highlighting the importance of inter-organellar cross talk during mitochondrial fission events (Ishihara et al., 2009). Mfn1 and 2 are responsible for fusing OMMs and tethering the mitochondria to the SR for Ca^2+^ signaling, making mitofusin proteins indispensable to inter-molecular and inter-organellar interactions (Chen et al., 2012). These proteins also have an important role in mitochondrial quality control by mediating fusion, guiding protein folding, and preventing ROS-induced mitophagy (Shirihai et al., 2015; Song et al., 2015). Opa1 mediates inner mitochondrial membrane fusion and maintains cristae structure, which ensures proper ETC function (Varanita et al., 2015). This increase in cristae integrity can reduce ROS and prevent cytochrome c release, preventing and reducing mitochondrial dysfunction and apoptosis in highly-metabolic tissues, like the heart and brain (Ong et al., 2010; Varanita et al., 2015).

Abnormal fission and fusion leading to reduced cristae integrity and less functionally efficient morphology—overtly spherical or elongated—are known contributors to heart failure due to their effects on metabolism and apoptosis (Ong et al., 2010; Papanicolaou et al., 2012; Dorn, 2016). Mitochondrial fission opens the mitochondrial permeability transition pore (MPTP) which can result in cell necrosis or mitophagy if not properly managed, as seen in ischemic conditions (Parra et al., 2008; Shirihai et al., 2015; Song et al., 2015). Activation of GPER and ERα has been shown to preserve mitochondrial function and decrease mitophagy after ischemia reperfusion injury via MPTP signaling through MEK/ERK activation, thereby decreasing apoptosis (Feng et al., 2017; Mahmoodzadeh and Dworatzek, 2019). These data, again, suggest the estrogen has cardioprotective effects by preserving mitochondrial integrity and inhibiting apoptosis. Testosterone has also been shown to protect against myocardial infarction through the AMPK pathway, elevating PGC-1α and preserving mitochondrial integrity leading to decreased cardiomyocyte apoptosis, as demonstrated by rodent models (Witt et al., 2008; Wang F. et al., 2015). The ability of both estrogen and testosterone to activate PGC-1α in cardiac tissue has been extensively studied, and it is well-established that PGC-1α regulates the transcription of Drp1, Fis1, Mfn1, Mfn2, Opa1, and other important mitochondrial dynamic proteins (Table 1; Witt et al., 2008; Papanicolaou et al., 2012; Martin et al., 2014; Wang F. et al., 2015; Park et al., 2017). This therefore implies a potentially shared pathway for cardioprotection by estrogen and testosterone, but direct evidence has remained elusive. Adding to the complexity, direct regulation of mitochondrial dynamics by the sex hormones has been well established in brain, breast cancer, prostate cancer, and adipocyte models but more research is needed to better characterize the direct effects of estrogen and testosterone in modulating signaling in cardiac tissue as well as inter-organellar cross talk between the mitochondria and other cellular organelles (Sastre-Serra et al., 2013; Capllonch-Amer et al., 2014; Lejri et al., 2018; Lee et al., 2020).

## Sarcoplasmic Reticulum and Mitochondrial Cross Talk

The K_ATP_ channel, found on both the mitochondria and the sarcoplasmic reticulum (SR), alters the electrochemical gradient through an influx of K^+^ into each organelle (Ranki et al., 2002; Er et al., 2004; Ballantyne et al., 2013; Gao et al., 2014; Bayat et al., 2016; Sakamoto and Kurokawa, 2019). In the mitochondrion, this change in K^+^ concentration causes an increase in K^+^/H^+^ antiporter activity, inducing an efflux of H^+^ ions from the mitochondrial intermembranous space. The resulting decreased membrane potential impairs organelle efficiency and reduces mitochondrial production of ATP. The activation of the K_ATP_ channel by both estrogen and testosterone has been shown to be cardioprotective in models of ischemia reperfusion injury (Er et al., 2004; Ballantyne et al., 2013; Gao et al., 2014; Sakamoto and Kurokawa, 2019). Estrogen, but not testosterone, can also regulate the SR K_ATP_ channel, which has also been shown to preserve cardiac function after ischemia reperfusion injury (Ranki et al., 2002). Evidence further suggests that testosterone may have a down-regulation effect on SR K_ATP_ channels in exercise models, suggesting a potentially antithetical effect from estrogen (Bayat et al., 2016). GPER activation has also been indicated as a possible mitigator of cell death during reperfusion injury through modulation of mitochondrial integrity, further supporting estrogen’s cardioprotective properties (Lee et al., 2012; Feng et al., 2017; Groban et al., 2019). Taken together, these data suggest potential mechanisms of cardioprotection via sex hormone regulation of K_ATP_ channels on mitochondria and sarcoplasmic reticula.

Another important ion to consider in this interorganellar crosstalk is calcium (Ca^2+^). Ca^2+^ is a divalent ion, and an essential mineral vital for cellular signaling, which has been a critical area of study for several decades (Berridge, 2003; Brookes et al., 2004; Clapham, 2007). Calcium signaling has also been extensively studied in the mitochondrion and plays important roles in the regulation of many enzymes in the Krebs cycle, electron transport complexes such as ATP synthase, as well as many other enzymes (Das and Harris, 1990; McCormack et al., 1990; Consolini et al., 2017). In cardiomyocytes, an essential mechanism of cardiac function is the maintenance of high levels of ATP in order to properly activate SERCA—a Ca^2+^ pump on the surface of the sarcoplasmic reticulum—and induce the reloading of Ca^2+^ in action potentials (Rosano et al., 1999; Piquereau et al., 2010; Witayavanitkul et al., 2013). Modulation of SERCA levels or activity and Ca^2+^ burden by testosterone is suggested to be cardioprotective during ischemic events (Witayavanitkul et al., 2013). Additionally, it has been shown that estrogen can increase SERCA protein expression, particularly SERCA2b, which causes a decrease in intracellular Ca^2+^, thus increasing cell survival in coronary arteries (Witayavanitkul et al., 2013; Hill and Muldrew, 2014; Groban et al., 2019).

In cardiomycocytes, move phrase to after (CICR), the primary driver of Ca^2+^ release is through Ca^2+^ induced Ca^2+^ release (CICR). One example of a channel which exists in the inner mitochondrial membranes and the SR are the ryanodine receptors (RyR/mRyR), which are responsible for releasing intracellular stores of Ca^2+^ ions through CICR (Beutner et al., 2005; Altschafl et al., 2007; Gambardella et al., 2018). Leaky RyR on the SR has been directly implicated in heart failure, as excess cytosolic calcium is absorbed by mitochondria resulting in dysregulation and further RyR leak from oxidative damage generated by mitochondrial ROS (Santulli et al., 2015). In contrast, when Ca^2+^ is released from inositol-triphosphate receptors (IP_3_R)—receptors responsible for Ca^2+^ release and a mechanism for triggering CICR—and absorbed by mitochondria, whose function, mitochondria or IP3R and ATP production increases; however, when mitochondria number is compromised—such as in heart failure, the Ca^2+^ release can induce arrhythmias (Hohendanner et al., 2015; Seidlmayer et al., 2016). The effect of estrogen on RyR expression and function is mixed and poorly understood, whereas growing evidence is implicating testosterone in increasing expression and function (Tsang et al., 2009; Hsu et al., 2015; Evanson et al., 2018; Groban et al., 2019; Mahmoodzadeh and Dworatzek, 2019; Jiao et al., 2020). For IP_3_R, the role of estrogens is even more poorly understood, with some work implicating estrogen’s ability to activate IP_3_ production in liver and smooth muscle cells, whereas testosterone has been shown to directly trigger IP_3_ production and IP_3_R activation in cardiomyocytes (Marino et al., 1998; Vicencio et al., 2006). In summary, these data suggest that the sex hormones play an important role in regulating intracellular Ca^2+^, itself a regulator of cellular apoptosis through the mitochondrion, thereby highlighting an additional cardioprotective role of the sex hormones (Pinton et al., 2008; Groban et al., 2019).

## Cell Death

There is conflicting information regarding the role of the sex hormones in regulating cell death in cardiac tissue, and therefore, indicated a need for further research in this area (Djouadi et al., 1998; Morris and Channer, 2012; Hsieh et al., 2015; Wang F. et al., 2015; Gagliano-Juca and Basaria, 2018; Jones and Kelly, 2018). Cell death pathways, including apoptosis, autophagy, necrosis, and pyroptosis, have been implicated in inducing cell death in CVD among various cell types within the heart including cardiomyocytes, endothelial cells, and monocytes/macrophages (Subramanian and Shaha, 2007; Wang F. et al., 2010; Chen et al., 2014; Feng et al., 2017; Amgalan et al., 2020; Di Florio et al., 2020). In macrophages, estrogen has been shown to regulate intracellular Ca^2+^ levels, which modulates Bcl-2 activity, and decreases Bax translocation to the mitochondria, thereby preserving cell viability through inhibition of intrinsic apoptosis (Subramanian and Shaha, 2007). Preliminary data in cardiomyocytes has shown that estrogen regulates Akt through ERα which attenuates ROS-induced intrinsic apoptosis in female mice, but not males (Wang F. et al., 2010; Hevener et al., 2020). While the previous study determined ERβ does not play an anti-apoptotic role in response to ROS, estrogen signaling through ERβ has been shown to decrease cardiac apoptosis by increasing mitochondrial Complex IV in rodent trauma-hemorrhage models (Hsieh et al., 2006). However, more recent studies have indicated that ERβ is not highly expressed on cardiomyocytes, and therefore may not play a major cardioprotective role in CVD (Pugach et al., 2016; Groban et al., 2019).

Testosterone has been shown to indirectly regulate necrosis of cardiomyocytes through NF-κB apoptotic signaling pathways, however, more studies on the regulation of cell death by testosterone are essential to better understand its role in CVD (Xiao et al., 2015). Estrogen and testosterone’s roles in apoptosis may be altered according to receptor expression and cell type (Pugach et al., 2016). Therefore, further studies are necessary to better understand how signaling via both estrogen and testosterone influence cardiac apoptosis in CVD. Necrosis and pyroptosis are inflammatory cell death pathways regulated via caspase enzyme activity, which is induced when irreversible damage occurs in the tissues (Bergsbaken et al., 2009; Gao et al., 2019; Zhaolin et al., 2019). These pathways, more specifically, are initiated when the MPTP is damaged or uncontrolled in mitochondrial fission, as seen in CVD (Song et al., 2015; Amgalan et al., 2020). Estrogen treatment has been shown to inhibit necrosis and induce apoptosis in both sexes with lupus nephritis (Jog and Caricchio, 2013). In general, research on pyroptosis is limited in cardiac tissue and work has yet to indicate a direct role of the sex hormones.

## Conclusion

**FIGURE 1:**
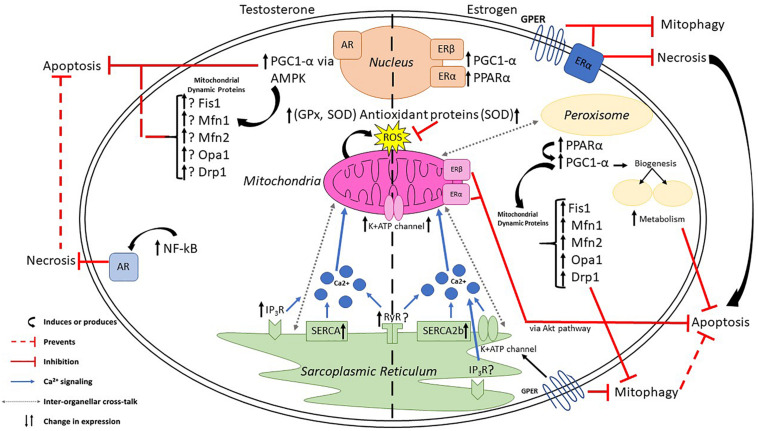
Estrogen and testosterone influence mitochondrial dynamics and cross talk between organelles. Estrogen, via PPARα, upregulates PGC-1α to initiate transcription of mitochondrial dynamic proteins and induce peroxisomal biogenesis. Testosterone upregulates PGC-1α, via AMPK, to influence mitochondrial dynamics. Both sex hormones increase antioxidant proteins to mitigate damaging reactive oxygen species, regulate Ca^2+^ signaling via K^+^ATP channels on the sarcoplasmic reticulum, and prevent various forms of cell death in CVD including apoptosis, necrosis, and mitophagy.

There are clinical disparities in CVD risk and incidence, which could be caused by known sexually dimorphic differences in cardiac cells and tissues. These differences are driven by the sex hormones—estrogen and testosterone—and the presence of their receptors ERα, ERβ, GPER and AR, which are expressed differentially in varying organ systems and cell types. Studies have shown that both estrogen and testosterone can directly regulate mitochondrial biogenesis, ROS production, inter-organellar interactions between the mitochondria and the endoplasmic reticulum, in addition to preserving cell viability (Figure 1). Nevertheless, further studies are needed to better understand the exact mechanism of each sex hormone in regulating mitochondrial dynamics, specifically the regulation of mitochondrial fission and fusion proteins, so to establish differential function of each and elucidating the cause of CVD disparities between the sexes. Research on the cardioprotective effects of sex hormones has predominantly focused on estrogen, leaving much to be studied regarding testosterone’s regulatory function in CVD. This review hopes to inspire others to begin focusing on the regulatory role of sex hormones in mitochondrial function and dynamics, as well as inter-organellar cross talk.

## Author Contributions

SL wrote the mitochondrial dynamics section and metabolism, edited the manuscript, and created the Figure 1. JB wrote the section on cell death and Ca^2+^ regulation, created the Table 1, and reviewed the manuscript. MS compiled the sources for metabolism and dynamics, reviewed the manuscript, table, and figure. SG-M wrote the introduction and conclusion, edited the text, figures and table, and reviewed the final manuscript. All authors contributed to the article and approved the submitted version. MRS Co-wrote metabolism and Ca^2+^ sections, edited text, and reviewed the manuscript, table, and figure.

## Conflict of Interest

The authors declare that the research was conducted in the absence of any commercial or financial relationships that could be construed as a potential conflict of interest.
